# SpaceOAR Complication Affecting the Treatment of Prostate Cancer

**DOI:** 10.7759/cureus.58485

**Published:** 2024-04-17

**Authors:** Jacfar Hassan, Aria Kieft, Steven Miller

**Affiliations:** 1 Radiation Oncology, Wayne State University School of Medicine, Detroit, USA; 2 Radiation Oncology, Detroit Medical Center - Wayne State University, Detroit, USA

**Keywords:** delay of care, complication of treatment, sbrt (stereotactic body radiotherapy), : prostate cancer, spaceoar hydrogel

## Abstract

A 60-year-old male presented with an elevated prostate-specific antigen (PSA) of 10 ng/ml. A transrectal ultrasound-guided prostate biopsy showed prostate adenocarcinoma GS 4+3 (grade 3) with 5 out of 12 cores positive for malignancy. He initially planned to have prostate stereotactic body radiation therapy (SBRT) with SpaceOAR gel insertion in his rectoprostatic space to reduce radiation to the rectum. Magnetic resonance imaging (MRI) two months after SpaceOAR insertion showed evidence of infiltration of the SpaceOAR within the anterior rectal wall. This delayed his treatment and he was started on a short course of androgen deprivation therapy with Leuprolide while waiting for absorption of the gel. After completion of androgen deprivation therapy, the patient was treated with external beam radiation therapy (EBRT) to the prostate, seminal vesicles, and pelvis to a total dose of 6000 centigray (cGy) in 20 fractions at a dose per fraction of 300 cGy. He did well after treatment with minimal side effects.

## Introduction

Prostate cancer is the most common cancer in men, with more than 268,000 new cases diagnosed annually in the United States, and the second leading cause of death after lung cancer ​[1]. Patients with localized prostate cancer can choose between strategies such as active surveillance, surgery, and radiation therapy with or without hormone therapy ​[2]. The rectum is anatomically adjacent to the prostate, which puts it at risk for damage from radiation during radiation therapy for prostate cancer, and limits the dosage of radiation needed for adequate treatment of prostate cancer. Therefore, the rectum is commonly referred to as a dose-limiting structure. Radiotherapy is associated with 5% or lower grade three-toxicity rates and grade four-toxicity rates of less than 1% ​[3]​. While the rate of severe toxicity is low, it can be life-altering for the patients who experience it. Multiple techniques and products were developed to displace the rectum away from the prostate to decrease the risk of radiation injury to the rectum. One of these is SpaceOAR, which is a hydrogel consisting of water and polyethylene glycol. It is injected transperineally as two precursor fluids between the anterior rectal wall and prostate fascia ​[4]​. As the two fluids mix, they rapidly expand and reach a firm gel-like consistency in the adipose tissue in this space. The hydrogel maintains its shape for approximately three months, after which it undergoes hydrolysis, is absorbed by the body, and is eliminated renally. While the SpaceOAR generally benefits patients by reducing the radiation dose to the rectum, there are some potential complications. These complications include rectal wall infiltration, rectal perforation, periprostatic venous intravasation, or prostatic abscess ​[4]​. Here, we report a case of rectal wall infiltration of SpaceOAR. 

## Case presentation

A 60-year-old male presented with an elevated prostate-specific antigen (PSA) of 10 ng/ml. The patient endorsed daytime frequency, urgency, occasional weak stream, and nocturia, but denied any dysuria or hematuria. A transrectal ultrasound-guided prostate biopsy demonstrated prostate adenocarcinoma GS 4+3 (grade 3) in 5 out of 12 cores. Various treatment options, including hormonal therapy, radiation therapy, radical prostatectomy, and ablative therapy, were discussed with the patient. He declined surgical resection and wished to proceed with primary radiation therapy to the prostate. Various radiation options were presented to the patient, including external beam radiation therapy (EBRT - typically delivered in 4-6 weeks of daily radiation treatments, Stereotactic Body Radiation Therapy (SBRT - typically delivered in 5 high-dose treatments every other day), and brachytherapy (radiation delivered from inside the prostate by an implanted radioactive source). The patient decided to have prostate SBRT treatment. Before beginning his SBRT treatment, he underwent an MRI of the pelvis, which demonstrated organ-confined disease. There were dominant nodules in the right posterior base and bilateral mid-posterior lateral peripheral zone, with an overall Prostate Imaging-Reporting and Data System (PI-RADS) score of 4/5. He also underwent SpaceOAR gel insertion. 

**Figure 1:**
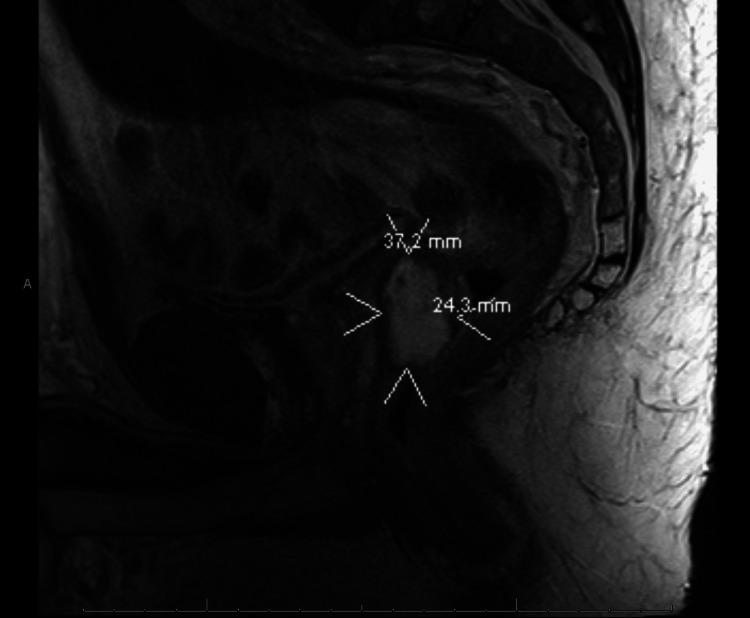
T2-weighted sagittal MRI of placement of SpaceOAR hydrogel in rectoprostatic space

**Figure 2:**
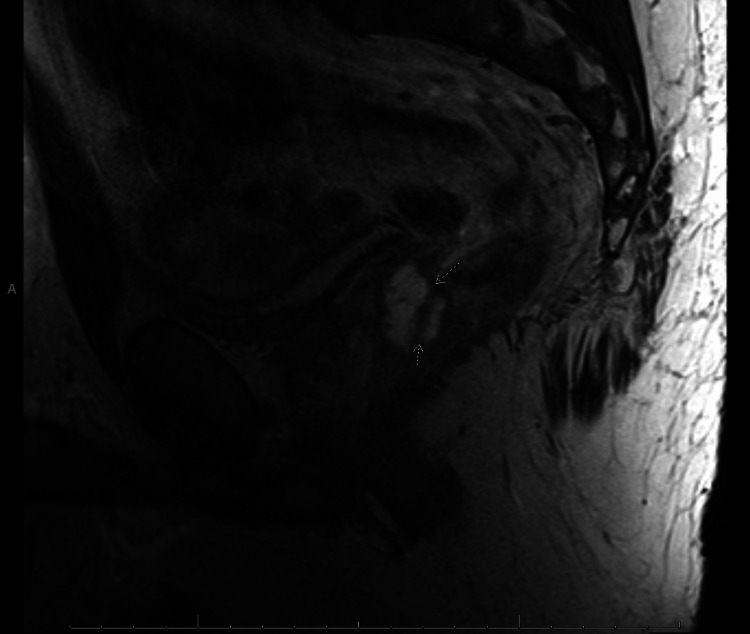
T2-weighted sagittal MRI (image taken laterally to Figure 1) with arrows indicating spaceOAR (hypointense) dissection of the rectal wall (hyperintense line)

**Figure 3:**
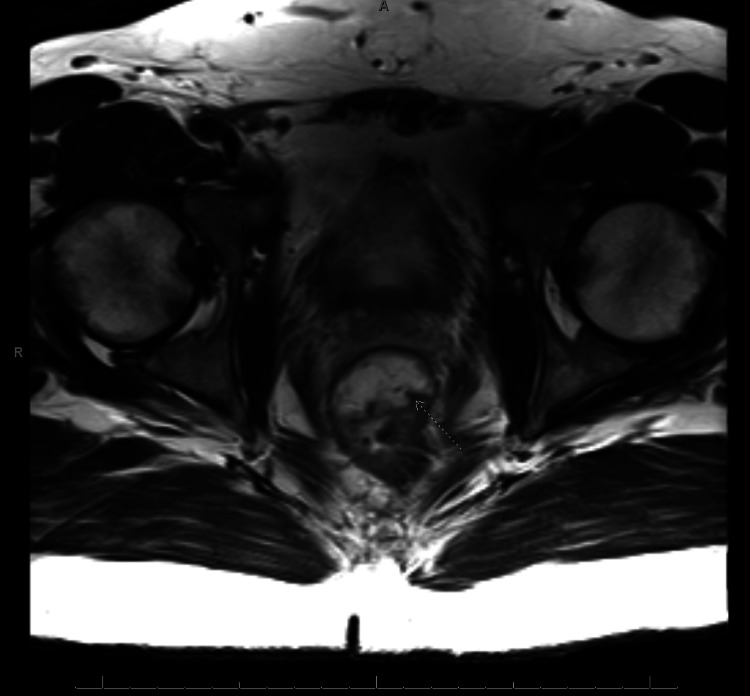
Axial T2 weighted MRI of the spaceOAR (hypointense) with arrow indicating disruption of the anterior rectal wall.

The gel was deposited in the rectoprostatic space. A colonoscopy a month after SpaceOAR insertion demonstrated an unremarkable perianal examination. However, there was a localized area of mildly erythematous mucosa found in the rectum in the region of the submucosa and rectum just beyond the anal verge, likely due to the spacer. Two months after SpaceOAR insertion, a repeat MRI showed evidence of infiltration of the SpaceOAR within the anterior rectal wall, with delamination of the muscularis propria (Figures 1-3), resulting in the accumulation of spacer material in the wall. The SBRT did not proceed as scheduled due to the suboptimal positioning of the spacer. The recommendation was for the patient to wait for the absorption of the spacer. He started a short, three-month course of androgen deprivation therapy with Leuprolide while awaiting spacer absorption to prevent disease progression. 

**Figure 4:**
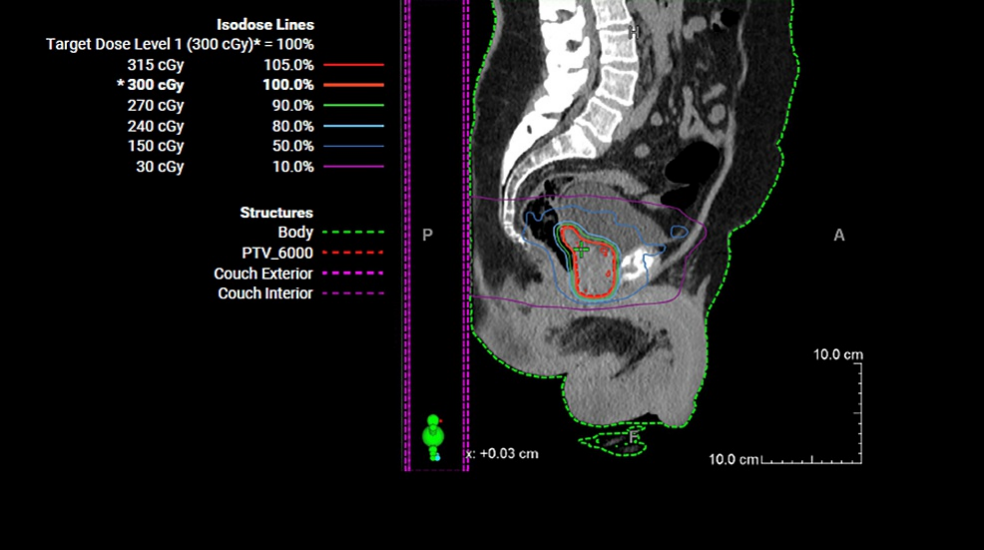
Sagittal CT of the treatment plan for each fraction of radiation to the prostate; 300 cGy in 20 fractions

After spacer absorption, the patient was treated with external beam radiation therapy (EBRT) to the prostate, seminal vesicles, and pelvis to a total dose of 6000 centigray (cGy) in 20 fractions at a dose per fraction of 300 cGy (Figure 4). The patient tolerated the treatment well. He was seen for a follow-up visit a month after cessation of EBRT. At that time, he reported urinary frequency, dysuria, and nocturia. He reported nocturia two to three times a night. He continued to take cranberry juice and Azo for his dysuria. He denied any history of hematuria, postvoid dribbling, and urinary incontinence. Using the the Common Terminology Criteria for Adverse Events (CTCAE v5) this was classified as grade I noninfective cystitis. [5] He also denied any tenesmus, abdominal pain, or rectal bleeding. He reported no history of erectile dysfunction but mentioned that his erections were not adequate at times. He was given a prescription for Viagra. The patient's PSA was found to be 6.438 ng/ml. He was scheduled for a follow-up appointment in three months. 

## Discussion

Radiation therapy for prostate cancer puts the rectum at risk for significant damage due to its proximity to the prostate. One method of protecting the rectum from radiation damage is rectal hydrogel spacers such as SpaceOAR. These spacers increase the distance between the prostate and rectum, significantly decreasing the rectal radiation dose and toxicity rate, and showing improvements in bowel, urinary, and sexual QOL ​[6]​. The gel is injected through the perineum into the fatty space between the anterior rectal wall and Denonvilliers' fascia ​[4]​. 

FDA approval for SpaceOAR was based on a prospective randomized single-blinded clinical trial in which 222 men with early-stage, low- to intermediate-risk prostate cancer planning to undergo radiation therapy were randomized to spacer hydrogel injection or no injection ​[7]​. The patients were followed for three years. The procedure to place the spacer device was typically tolerated well and was placed correctly 99% of the time ​[7]​. The investigators found that patients with the spacer had less late rectal toxicity and reported better quality of life in bowel and urinary symptom categories six months after treatment. Also, men who had potency at baseline reported higher rates of "erections sufficient for intercourse" ​[7,8]​. While the SpaceOAR generally benefits patients by reducing the radiation dose to the rectum, there are potential complications such as rectal wall infiltration, rectal perforation, periprostatic venous intravasation, or prostatic abscess ​[4]​. 

Careful surveillance of postmarket SpaceOAR data is essential. Two recent reviews of the "manufacturer and user facility device experience database (MAUDE)" demonstrated that while rare (around 9 in 1000 spaceOAR cases), 54.1% of patients with adverse events were symptomatic [9]. In 15.7% of cases, the complications led to a delay or change in the radiation treatment plan [9]. The most cited adverse event was mispositioned gel (57%), usually found on post-injection imaging to infiltrate the rectal wall ​[2]. Infection after SpaceOAR was documented in 17.6% of cases, and rectal ulceration in 10.5%. Three (0.5%) SpaceOAR placement complications resulted in death, though the exact cause was not reported in this article ​[2]​. 

To understand the range of presentations experienced by patients with mispositioned spacers, we reviewed case reports and will summarize three of them here: In one case report by Mclaughlin et al., a man underwent high-dose stereotactic ablative radiation therapy (SAbR) to the prostate without androgen deprivation therapy after apparently uncomplicated SpaceOAR placement. Five months after the completion of radiation therapy, he was found to have an extensive anterior rectal wall ulceration. There were no diffuse radiation changes in the adjacent rectum. He required a diverting colostomy. Hyperbaric oxygen therapy was attempted to improve healing. Unfortunately, he developed a significant infection, eventually requiring abdominoperineal resection fifteen months after radiation therapy [10]. 

In another case reported by Kuperus et al., a man underwent space OAR placement to treat his prostate cancer with definitive radiation and androgen-deprivation therapy (ADT). Shortly after the procedure, he had severe pain in the rectum and buttocks. Three weeks later, he was diagnosed with rectourethral fistula. As he was no longer a candidate for radiation, he underwent a robotic prostatectomy and repair of the rectal defect with diverting loop ileostomy [11].

Finally, in a case report published by Boerkamp et al., a man underwent a colonoscopy a week after spacer placement due to a positive occult blood test. A polyp was removed from the cecum, but no other abnormality was noted. He proceeded with radiation as planned, but the spacer was no longer visible on daily imaging by the 12th fraction (of 20 planned fractions). An MRI demonstrated a new sinus tract between the rectoprostatic angle and the inferior rectum through which the spacer was assumed to have extravasated. No additional radiation was delivered, and a coloscopy showed a healing sinus. The patient was managed conservatively, and they decided to monitor PSA rather than proceed with any other prostate cancer therapy [12]. 

## Conclusions

When discussing the risks and benefits of prostate cancer-directed radiotherapy with our patients, it is essential to thoroughly review the possible complications of each part of the proposed therapy. Understanding the risks of any medical intervention empowers patients to decide what is best for their cancer journey. When discussing SpaceOAR placement, patients should be informed that procedure complications are rare. However, risks include both asymptomatic and symptomatic misplacement of the device that can delay radiotherapy or cause similar toxicity to that which the device is aimed to avoid. With continuing improvement in radiation delivery techniques, choosing to utilize a hydrogel spacer should be highly individualized.
